# Supplementary material for: Are carbon emissions trading and green financial instruments synergistic? -Comprehensive quantitative research based on content analysis
Source: PLoS One. 2024 Mar 7;19(3):e0298601. doi: 10.1371/journal.pone.0298601 (PMC10919601; doi:10.1371/journal.pone.0298601)
Supplement: S1 Data — (ZIP) [file pone.0298601.s001.zip › Data/地级市Z_F工作报告.zip!/║ú─╧/╚2╤╟╩╨/└┤╫╘╣1⁄2╓┌║┼í╛┬φ┐╦╩2╛▌═°í┐_2008.docx]

各位代表：

现在，我代表市人民政府向大会作工作报告，请予审议，并请市政协委员和其他列席人员提出意见。

2007年工作回顾

2007年是新一届政府的开局之年，一年来，在邓小平理论和“三个代表”重要思想的指引下，在党的十七大胜利召开的鼓舞下，在省委、省政府和市委的正确领导下，在市人大、市政协的监督和支持下，市政府紧紧团结和依靠全市人民，牢固树立和认真落实科学发展观，全力打造“五个三亚”，着力推动三亚又好又快发展，圆满完成了市五届人大一次会议确定的各项年度任务，为胜利实现本届政府的各项工作目标奠定了良好的基础。

一、全力打造实力三亚，城市综合实力显著提升

――经济保持高速增长。2007年全市生产总值完成122.3亿元（不含农垦，下同），同比增长22.1%，增速同比提高7.4个百分点，为1994年以来的最高水平；其中，一、二、三产业分别实现增加值22.9亿元、48.4亿元、51.0亿元，同比分别增长9.9%、34.4%、22%。全市地方财政收入22.1亿元，同比增长118.9%；其中，一般预算收入15.4亿元，增长58.4%。全市完成固定资产投资109.7亿元，同比增长59.9%。至去年底，全市各项存款余额222.9亿元，比年初增长36.2%；城乡储蓄存款余额94.7亿元，增长15.2%；金融机构各项贷款余额93.2亿元，增长22.6%。

――产业结构进一步优化。旅游业等传统优势产业发展迅速，商贸产业等新兴产业发展加快。旅游业方面，南山、大小洞天旅游区成为首批国家5A级旅游景区，凯宾斯基等一批度假酒店已投入试营业。凤凰机场扩建工程顺利推进，国际航站楼项目已建成正式运营；新开了英国曼彻斯特――三亚包机、俄罗斯新西伯利亚――三亚航班，三亚直飞东南亚各国的航路调整顺利实现；2007年凤凰机场旅客吞吐量已达到531万人次。三亚至广州、上海、北京的列车已开通，凤凰岛国际客运港已正常通航。积极开展“国际旅游年”各项工作，“清凉一夏，三亚度假”夏季旅游宣传促销等活动引起了强烈反响。2007年全市旅游饭店（酒店）接待过夜游客538.4万人次，同比增长18.4%；其中国际游客52.2万人次，增长34.3%。旅游收入80.1亿元，同比增长22.5%；其中旅游外汇收入2.4亿美元，增长43%。农业方面，虽然遭受了旱灾和台风袭击，但由于抗灾措施到位，及时引导农民调整种植结构，农业生产仍然保持了平稳增长。农业完成总产值35.1亿元，同比增长10.5%。热带水果、热带花卉、冬季瓜菜、海洋渔业等特色优势产业持续较快发展，农业结构日趋优化。工业方面，实现增加值27.3亿元，同比增长16.2%。混凝土、水产品加工、船舶修造等行业的较快增长成为支撑工业增长的主要因素。工业园区规划建设顺利推进，创意新城开发建设已正式启动。房地产业方面，加大了土地储备力度，加强了土地资产管理，至去年底，政府土地储备库储备量已达1.8万亩。房地产投资、建设、销售均保持健康发展势头。商贸产业方面，城乡流通网络不断完善，具有旅游特色的商业网点体系初步形成。在旅游业等产业快速发展的带动下，商贸产业蓬勃发展，全年实现社会消费品零售总额29.4亿元，同比增长22.8%。

――城市建设不断加快。完成了三亚市城市总体规划重大调整、三亚市旧城区控制性详细规划、海棠湾分区规划及城市设计。火车站临时出口路、鹿回头半岛基础设施、城市夜景灯光工程等项目已完成，三亚湾新城、凤凰国际水城、时代海岸、新佳旅业鹿回头项目、鹿回头爱地项目、三亚悦榕庄以及东环铁路三亚段等项目建设进展顺利，三亚绕城公路亚龙湾及天涯连接线、东线高速公路海棠湾出口路、勤政路、红树林公园等项目已开工建设。新开通了4条城市公交线路和2条农村公交线路，公交候车亭及站牌建设正在推进，公交网络进一步完善。

――新农村建设顺利推进。进一步增加“三农”投入，2007年财政预算安排用于“三农”的支出达到2.8亿元，预算外还安排了新农村建设资金5340万元和设施农业资金5000万元。大力调整农业结构，积极发展无公害农业，加快了供港澳果蔬基地建设。把“设施农业+产业化”作为发展现代农业的一个重要抓手，进一步加大科技兴农力度，扶持设施农业集约化发展，目前全市设施大棚面积已达到12150亩。积极探索“龙头企业（行业协会）+农民专业合作社”等合作模式，已成立50家农民专业合作社。大隆水库主体工程已完工，水库移民安置工程已通过国家竣工验收；抱古水库除险加固、赤田水库应急加固和大隆灌区工程等水利设施建设顺利推进，农村生产条件进一步改善。大力推进文明生态村示范点建设，农村公路“通达工程”8项89公里已基本完成，“通畅工程”25项143公里仍在积极推进，43条新农村进村主干道共54公里已基本完工，全市完成“一池三改”沼气池建设3600户，完成了36宗饮水安全工程建设，完成民房改造800余户，农村民主法制建设进一步加强，农村面貌焕然一新。

二、全力打造活力三亚，发展动力不断增强

――对内改革进一步加快。成立了海棠湾开发建设有限公司和三亚湾开发建设有限公司，继续推进国有劣势中小企业退出国有序列工作，国企改革工作取得新进展。进一步调整和优化财政支出结构，不断增强统筹保障能力和服务民生能力，加大了新农村建设、社会低保以及教育、医疗等社会事业投入力度。出租车行业改革初见成效，单车承包租金全面下调。预算管理制度改革等财税改革、教育人事制度改革、农垦经济体制改革以及事业单位改革等也取得新进展。

――政府自身改革顺利推进。进一步加强执行文化建设，积极组织开展创建“五星级服务机关”等活动，大力推行首问责任制、岗位责任制和跟踪督办制，建立行政过错追究制度和投诉处理机制，行政效能不断提高。大力贯彻实施《行政许可法》，进一步加强规范性文件制订、行政复议等工作，积极推进行政执法体制改革，法治政府建设得到加强。加大了审计监督力度，完成180个审计项目，审计处理各类违规资金1.5亿元，为财政增收节支3.1亿元。依法接受人大监督和政协监督，认真办复市人大代表建议99件和市政协委员提案120件，人大代表和政协委员的满意率达到96%；加强与各人民团体的联系，虚心听取各界人士的建议、意见；开通了“政风行风”热线，主动接受社会各界监督。进一步加大行政监察工作力度，大力查处行政不作为、乱作为等违法乱纪行为，深入开展反腐斗争，查办各类违法违纪案件26宗，为国家和集体挽回经济损失1056万元。

――对外开放取得新成效。大力开展对外交流，与夏威夷州毛伊县、黑龙江省伊春市等结为友好城市。积极组织有关部门和企业参加第四届“珠洽会”等商务活动，在项目洽谈、投资推介、经济交流和合作等方面取得积极成效。2007年实际利用外资1.3亿美元，同比增长18.2%。海棠湾首期8宗共2399亩用地公开招标出让，引进8家大企业，盘活了该片区闲置用地，11家高星级酒店建设前期工作正紧锣密鼓地开展，片区主要基础设施建设已铺开。继续扶持出口企业实行技术改造，全市完成进出口总值6650万美元，同比增长13%；其中，出口5700万美元，增长39%。

三、全力打造美丽三亚，生态环境质量保持全国一流

――节能减排工作稳步推进。红沙污水处理厂升级改造、鹿回头区污水处理、三亚湾新城污水处理等污水处理项目以及生活垃圾填埋场、亚龙湾冰蓄冷区域供冷系统等项目已动工。积极开展建筑节能示范工程建设，加强了建设项目的环保审批以及对重点污染源的监控工作。拆除了耗能大户三亚华盛天涯水泥厂，使2007年全市能源消费总量减少1.8万吨标准煤，拉动全市万元GDP能耗下降0.3%。继续落实排污许可证管理制度，对违法排污企业进行严厉查处。

――“生态市”建设不断加快。积极开展环保专项行动，加大了海洋环境监察工作力度，并以三亚湾为重点，对各大海湾进行全面整治。加快实施海防林恢复工程，积极推进退耕还林、退塘还林、退果还林、营造“三边”防护林以及城市绿化等工作，2007年末全市森林覆盖率达到66%，城市建成区绿化覆盖率44.9%，绿地率41.3%，人均公共绿地面积18.9平方米。生态环境质量继续保持全国一流，被评为中国十大休闲城市、最佳度假目的地。

――城市管理进一步加强。围绕“双提高”大行动，进一步加强城市管理，推行“综合行政执法＋属地联动管理”工作方式，积极开展专项整治行动，依法拆除违章建筑16万多平方米，取缔乱占乱摆摊点5600多个，查扣非法营运摩托车、人力车4800多辆，查处违法经营网吧56家（次），取缔无证照废品收购站36家，取缔生猪私宰窝点17家。积极开展创建国家卫生城市活动，城市环境卫生大幅改善，已两次通过省爱卫会工作检查，去年12月底又通过了国家爱卫办的暗访检查。

四、全力打造文化三亚，城市品位进一步提升

――文化产业方兴未艾。进一步扩大和深化城市名片成果，“六个一工程”大型系列文化活动顺利推进，三亚首部风光故事片《一座城市和两个女孩》已经公映，超级美秀《浪漫天涯》大型演艺已经正式上演。积极引进有实力的文化产业企业和有特色的影视、传媒、文化、艺术产业项目，南中国海影视文化生态园已开工建设。继续实施大型文化节庆赛事活动牵引发展战略，举办了世界先生总决赛、第57届世界小姐总决赛、新丝路模特大赛、第三届国际电视广告艺术节、第二届中国（三亚）国际热带兰花博览会等重大文化节庆赛事活动，为文化产业发展创造了良好氛围。

――文化事业蓬勃发展。文化体制改革取得实质性进展，吸引社会资金建成了古生物化石博物馆，积极推动农村文化阵地建设，六镇的综合文化中心已建成。继续推进广播电视村村通工程，全市广播电视人口覆盖率达95%，有线数字电视转换工作顺利开展。积极挖掘历史文化、民族文化以及民俗文化，进一步加强文物保护工作，崖城镇被评为“中国历史文化名镇”。群众广场文化活动、社区文化活动、文化下乡活动和全民健身运动蓬勃开展，成功举办了“2007中国三亚三月三民族风情节”等活动。进一步加强文化市场管理，文化市场清理、检查、整顿工作取得新成效。

五、全力打造和谐三亚，人民生活大幅度改善

――人民生活水平明显提高。2007年城镇新增就业岗位19880个，农村富余劳动力转移15100人，城镇登记失业率控制在2%以内。城乡居民收入保持快速增长，城镇居民人均可支配收入11642元，同比增长23.6%，扣除价格上涨因素，实际增长18.2%；农民人均纯收入4376元，增长17.5%，扣除价格上涨因素，实际增长10.6%。不断强化社会保障工作，社保政策得到较好落实，社保基金监管进一步加强。2007年末基本养老保险覆盖率达到82%，城镇失业保险覆盖率达到75%。城镇居民医疗保险试点工作成效显著，参保率达95%，新型农村合作医疗参合率达98%。全市城乡低保工作顺利开展，基本实现应保尽保，低保标准进一步提高。推行廉租房和经济适用房工作取得新进展，居民住房条件进一步改善。

――社会事业全面发展。进一步加大了社会事业投入力度，2007年科技支出同比增长409.1%，教育支出增长50.1%，医疗卫生支出增长44.6%。积极推进三亚南繁科学技术研究院建设，进一步完善农业科技“110”服务体系，不断加强科技创新服务体系建设，加大科技人才引进力度，加快政务门户网站集群建设，科技进步对经济增长的贡献率达到44.5%。积极推进三更小学等18所中小学校舍建设，博雅中学建成招生，全市义务教育阶段公办学校已实施“两免一补”政策，实行了中等职业技术教育“三免一补”政策。市委党校扩建项目竣工，市教师进修学校一期建成投入使用，市中等职业技术学校一期、三亚学院二期和三亚航空旅游职业学院二期工程基本建成并迎接新生入学，琼州学院三亚校区已开工建设。市中医院新区门诊楼基本建成，市人民医院医技和外科住院楼正在进行装修，行政村卫生室覆盖率达到100%。出生人口性别比和人口自然增长率分别控制在112.6和9.5‰，完成年度计划指标任务。社会主义精神文明建设取得新成绩，社会治安综合治理成效显著，市场经济秩序持续好转，食品药品安全保障有力，信访、安全生产等工作进一步加强，社会政治保持稳定。国防动员、民兵预备役工作不断进步，拥军优属、拥政爱民活动深入开展，军政、军民团结进一步加强，荣获“全国双拥模范城”称号。民政、民族、宗教、人防、气象、防震减灾、工会、共青团、侨联、科协、妇女儿童、老龄、残联、新闻、统计、档案及修志等方面工作都取得了较大进步。

各位代表，一年来我市经济社会发展所取得的巨大成绩，离不开省委、省政府和市委的正确领导，离不开各个方面的关心、支持和帮助，是全市人民共同奋斗的结果。在此，我谨代表市政府，向在各个领域和岗位上辛勤劳动、做出贡献的全市人民，向给予我们支持与监督的人大代表、政协委员、各民主党派、工商联和无党派人士、各人民团体、社会各界人士，向大力支持我们工作的中央、省驻三亚单位、驻三亚部队、武警官兵和公安司法干警，向所有关心、支持三亚建设和发展的同志们、朋友们表示崇高的敬意和衷心的感谢！

回顾过去的一年，我们也清醒地看到，发展的道路上仍然存在许多困难和问题，人民群众还有一些不满意的地方，主要表现在：旅游产品结构单一，高消费精品不足；农业抗灾能力不强，农民持续增收困难较多；投资增长主要集中于房地产业和旅游业，投资结构有待进一步优化；城市建设仍需加快，管理水平亟待提高；劳动力供需结构性矛盾较为突出，就业培训压力较大；教育发展不均衡问题比较突出，农村学校建设步伐仍需加快；政府角色转变有待进一步加快，服务能力有待进一步增强。对这些问题，我们将予以高度重视，并采取有效措施在今后的工作中努力加以改进。我们真诚地期望各位代表、政协委员，各民主党派、工商联和无党派人士以及广大人民群众继续对我们给予批评和监督，帮助我们把政府工作做得更好。

2008年工作安排

2008年是深入学习贯彻党的十七大精神的重要一年，是海南建省办经济特区20周年，是实施“十一五”规划承上启下的关键一年。根据省委、省政府的总体要求和市委的部署，2008年政府工作的基本思路是：认真贯彻党的十七大精神，高举中国特色社会主义伟大旗帜，以邓小平理论和“三个代表”重要思想为指导，深入贯彻落实科学发展观，按照市第五次党代会的要求和部署，积极推进产业结构优化升级，继续加快改革开放，大力统筹城乡发展，进一步加强文化建设，着力改善民生，加强生态环境保护，推动三亚又好又快发展。

根据上述思路，综合考虑各方面的因素，2008年我市经济社会发展的主要预期目标是：地区生产总值增长13.5%；地方财政一般预算收入增长20%；固定资产投资达到135亿元，增长23%；全市旅游饭店（酒店）接待过夜游客增长12%；万元地区生产总值能耗下降2.3%；当年新增就业岗位15000个以上，城镇登记失业率控制在3%以内；城镇居民人均可支配收入增长9%,农民人均纯收入增长9%；人口自然增长率控制在9.5‰以内。为实现上述目标，2008年我们将重点抓好以下几个方面的工作：

一、推动产业结构优化升级，进一步增强城市竞争力

我们要在提升旅游产业等优势产业发展质量的基础上，积极促进文化产业、高科技创意产业等新兴产业发展，打造新的特色产业体系，加快转变经济发展方式。

――全力加快旅游产业国际化进程。以扩大对外开放为重点，积极探索建立与国际惯例和通行规则接轨的行业体制机制，进一步加快旅游国际化进程。一是积极推动投资主体国际化。以海棠湾等重点区域为平台，大力吸引有影响力的国际投资者，推动旅游项目投资主体国际化。加快与境外客源地国际旅游大集团的合资合作，积极鼓励和支持外商在我市依法设立独资、控股旅行社。二是积极推动旅游产品国际化。按照国际休闲度假旅游目的地的标准，打造独具特色的优质旅游产品：充分利用三亚高质量的热带海洋资源，深度开发潜水、海底观光、休闲渔业和海上游等海洋旅游产品，探索开发海上主题公园；继续发展高尔夫旅游，逐步壮大邮轮经济，培育游艇经济与会展经济；加快发展健康休闲旅游，启动羊栏和半岭温泉疗养度假区等项目建设，推进南新温泉康体疗养中心一期建设，创造条件打造规模大、档次高、有特色的中医保健和康复疗养中心；推进时代海岸、阳光海岸等项目建设，开发高雅健康的夜间娱乐项目，打造独具特色的集休闲、娱乐、餐饮、购物为一体的不夜城。三是积极推动旅游接待设施国际化。加快推进三亚华宇度假酒店、三亚悦榕庄、东方文华、鹿回头爱地等项目建设，力争已进入海棠湾的高星级酒店尽早开工。高标准修编景区规划，加快推进天涯海角、鹿回头等景区改造提升，加强南山、大小洞天国家5A级旅游景区建设，进一步完善亚龙湾国家旅游度假区建设，积极打造三亚湾旅游度假区，加快海棠湾“国家海岸”建设步伐，努力打造国际顶级的度假休闲区域。四是积极推动旅游服务管理国际化。大力开发地方特色旅游商品，积极引进世界名牌商品代理商参与免税店经营，构建商品类型丰富、功能完善、服务国际消费者的商业网络。积极促进银行、保险、证券、餐饮、交通运输、仓储物流、邮电通讯、信息咨询等现代服务业加快发展，完善综合服务功能。进一步加强口岸管理，提升口岸服务效率。加强从业人员培训，逐步建立健全国际化的道路标识系统、旅游标识系统、旅游咨询服务系统、旅游紧急救援系统。成立市旅游产业发展委员会，积极推进国家级旅游服务标准化试点城市建设，大力推行行业标准化管理，建立健全旅游行业协会。继续开展旅游诚信建设，进一步加大旅游市场整顿力度，不断完善旅游投诉和旅游联合执法机制。五是积极推动客源结构国际化。紧抓2008年奥运契机，精心策划旅游促销活动，同时充分利用国内外知名旅行商的营销网络，吸引境外高端游客。综合利用好落地签证、免签证等政策，尽量用足用活“一程多站”和航权开放政策，开通更多的国际直达航线航班，增强三亚的国际可进入性。

――促进房地产业理性发展。进一步加大土地储备力度，加强土地市场调控，坚持全面推行国有土地使用权“招拍挂”出让制度，合理配置土地资源。坚持两条路发展，一方面要继续发展旅游房地产，但必须进一步提高进入门槛，开发具有更高品位的休闲度假高端产品，推进三亚湾新城、凤凰国际水城、鲁能三亚湾度假区等项目建设；另一方面，要以货币补贴为主，以经济适用房和廉租房为辅的方式，保障三亚中低收入家庭住房。要加强对房地产市场的预测、监测，准确把握市场需求，调控开发总量，提升开发质量，保持房地产业健康发展。

――进一步壮大文化产业。继续落实《关于加快三亚文化产业发展的决定》，加快编制《三亚市文化产业发展规划》。为文化工作者创造良好的创作和生产制作环境，强化在职教育和在岗培训，努力提高文化产业从业人员的业务素质。加快推进体育中心、会展中心、三亚大剧院等基础设施以及南中国海影视文化生态园等产业项目建设，继续积极引进有实力、有特色的大型文化产业项目。全力组织好“六个一工程”大型系列文化活动，按照政府引导、市场运作、企业参与的原则，有选择性地引进、组织和培育精品文化节庆赛事活动。今年要举办好ELITE世界精英模特大赛、世界沙滩排球赛、国际铁人三项赛、2008奥运火炬传递等活动。

――积极培育高科技创意产业。全面推动创意新城基础设施建设，加快创意新城开发。积极引进有实力的企业和项目，加快推动与中兴公司的合作，促成中兴项目、丰华科技软件研发基地、蜗牛创意新社区、海云天网络以及中石油、中核建等企业如期开工，促进省微电子产业基地启动，大力引进芯片制造项目，推动南山电子信息产业基地建设，打造高新技术研发基地，形成高科技研发产业集群，促进产业结构实现跨越式发展。通过引进、孵化一批科技型、创意型项目，促进网络、动漫产业发展。促成同济科技园二期项目建设，尽快形成一批能见成效的产业项目。积极完善科技创新体系，鼓励企业创新，大力推进三亚南繁科学技术研究院、中国三亚农业科学城建设，加快推进城市信息化基础设施建设，积极推广信息技术应用，加强知识产权保护工作，为创意产业发展营造良好的环境。

――提升海洋产业竞争力。以优质鱼、虾、贝类和海珍品的精深加工为重点，积极鼓励和支持水产品加工出口龙头企业发展。加强水产品安全质量保障，打造“无公害”品牌。以加工业需求带动养殖业结构调整，加大优质水产品种的引进和推广力度，积极推进墨西哥湾扇贝养殖良种场等种苗工程项目建设。做好中心渔港评估和选址工作，加强渔业技术攻关与培训，继续采用“企业+渔民”的方式，以企业服务补给船为龙头，组织渔民跟帮作业，支持渔船更新改造，积极开发外海渔业资源。创造条件推动南海油气资源开发的补给、研发、储备和加工基地建设，发展相关工业。

――大力发展商贸产业。进一步加强商业基础设施建设，加快推进第一、二、三农贸市场以及鸿港市场改造升级。培育一批大型骨干流通企业，积极推行电子配送、电子商务等现代流通方式。培育新的消费热点，引导和促进居民消费规模扩大和消费模式升级，扩展信息、文化、健康保健、体育健身等新兴消费。继续推进“万村千乡工程”试点工作，积极发展农村连锁经营，进一步完善农村市场消费体系。加强市场监管，深入开展商贸行业市场秩序整顿，加大产品质量和食品安全整治力度，加快信用体系建设，营造和维护公平的市场竞争环境。力争2008年实现社会消费品零售总额35亿元，同比增长19%。

二、继续深化对内改革，积极扩大对外开放

我们要不断加快改革步伐，积极推进体制机制创新，进一步扩大对外开放，不断增强三亚发展活力。

――努力优化市场经济环境。充分发挥海棠湾开发建设有限公司和三亚湾开发建设有限公司的作用，进一步加强政府在整合城市资源方面的主导作用，搞好重点片区开发建设及配套基础设施建设，促进城市资源增值。稳妥推进市国资公司、市旅投公司、市旅游会展公司重组工作，完成市食品厂、市建材厂等一批劣势国企有序退出工作。建立健全国有资本经营预算制度，加强企业财务监管和风险控制，不断完善企业业绩考核体系和现代企业制度。实施中小企业成长工程，加快中小企业服务体系规划建设，探索建立中小企业信用担保基金，不断完善中小企业和非公经济的发展环境。积极建立行业协会运行机制，鼓励中介组织发展，发挥其在服务企业、行业自律、行业协调等方面的积极作用。

――继续推进财税金融体制改革。大力培植财源，提高房地产税等税收征管质量。进一步调整和优化财政支出结构，以保障民生为重点，促进支出向社会事业、新农村建设和基础设施建设等方面倾斜，同时充分发挥财税政策的导向作用，引导社会投资。建立预算绩效评价体系，加强财政绩效预算、绩效评价和绩效审计监督。强化国库集中收付管理，进一步完善财政投资评审制度，提高财政管理效能和财政支出效益。加强政府采购监管，建立政府债务风险监测和预警机制。支持金融体制改革，保持金融形势稳定，提高金融服务效率。此外，要贯彻落实中央和省农垦改革精神，支持国营农场加快改革步伐，提高农垦发展水平。

――不断提升招商引资质量。继续实施“大企业进入，大项目带动”发展战略，全力抓好招商引资工作，有效推进重点项目建设，在努力提升投资质量的前提下，促进投资高效增长。一是大力改善投资环境。进一步简化和完善投资审批制度，继续落实“一会五库”制度和项目进入联审制度，积极推行“代建制”。严格实行项目跟踪服务，完善项目风险预警机制，建立健全项目监督机制和退出机制。建立投资环境监督评议评价制度，逐步完善个人和企业的信用档案，形成社会信用体系基本框架。二是进一步扩大投资规模。坚持“三个坚定不移”的原则，千方百计推进重点项目建设，充分发挥其支撑作用。积极配合开行做好政府信用贷款项目的组织实施，争取开行对重大项目建设的信贷支持。抓紧开展有针对性的项目申报工作，争取国家、省更多资金支持。积极组织参加“珠洽会”、“广交会”以及海南省在香港等地举办的综合性招商活动，努力引进一批重大项目，做好协调服务，促进项目落地，提升项目履约率。三是注重提升投资质量。建立项目选择机制，强化产业政策引导，鼓励基础设施建设、农业综合开发、生态环境建设、高新技术产业等领域投资，尤其要注重生产型、税源型项目的引进。

――进一步扩大对外开放。积极开展与中国――东盟自由贸易区、泛珠三角、长三角、环渤海等区域的交流合作，创造条件吸引国内外知名企业和公司在三亚设立总部或研发基地。继续实施科技兴贸战略，支持出口企业技术改造，支持东吉公司、远跳公司、升达水产公司等龙头企业加快发展。重点发展反季节瓜菜、热带水果、热带花卉、海产品等热带优势农副产品及其深加工产品，加快海产品加工出口基地、花卉出口基地、瓜果菜出口基地等基地规划建设。实施出口品牌战略，鼓励企业扩大具有自主知识产权、自主品牌的商品出口。建立健全外贸预警机制，有效防范、规避和化解贸易风险。力争2008年实现外贸进出口总值7647万美元，其中出口总值6670万美元。

三、加快城市建设步伐，全力推动城市发展

经济发展促进城市发展，城市发展又反过来促进经济发展。我们要进一步拓展城市发展空间，加快基础设施建设，完善城市服务功能，提高城市管理水平。

――积极拓展城市发展空间。以规划为依据，加快完善“一城六镇”的城镇发展框架，推进六镇以及农场场部建设，特别是要全力加快海棠湾、红塘湾等重点区域开发建设进度，进一步拓展城市发展空间。把新区建设和旧城改造有机地结合起来，在搞好拆迁补偿，切实维护居民利益的前提下，进一步加大土地整理储备力度，抓紧时间尽快对旧城土地资源进行统一整合，积极推行土地成片规模开发，提高土地利用效率，缓解用地紧张，同时充分利用现有资金。要以项目建设为依托，结合城中村改造，推进阳光海岸、榕根、月川、临春、东岸、鹿回头等片区开发。

――加快重大基础设施建设。抓好凤凰机场扩建工程和南山货运港一期工程建设，力争南山货运港今年投入使用。重点推进东环铁路三亚段、东线高速公路海棠湾出口路、三亚绕城公路及其亚龙湾、天涯连接线建设；年内要完成鹿回头海景大道、金鸡岭路改造等工程建设；月川新城区道路、亨新大道等项目要开工建设。积极推进崖城水厂扩建工程，进一步扩大燃气设施覆盖范围，加快城乡电网改造。抓紧海棠湾基础设施建设，为区域开发提供保障。

――提高城市管理水平。建立健全城市网格化管理新机制，做到网格单元、岗位人员、目标责任一一对应，层层落实。进一步理顺行政执法体制，完善城市管理重心下移工作，积极探索建立疏堵结合的城市管理新模式。加快城市公共资源利用市场化步伐，推广公开招标拍卖出让，提高运营管理效率和效益。严厉打击违法买卖土地、违法建房等行为，加强规划监管力度，切实落实城镇居民和农民的住房报建制度，配合重点项目建设，加大对违法建筑的巡查和打击力度。继续大力发展公共交通，优化公交线路，进一步规范出租车运营秩序，不断加强道路交通整治，坚决打击非法营运车辆。加大创卫投入，针对暗访组提出的不足进行整改，继续深入开展环境卫生综合整治，落实好“门前三包”，精心打造创卫样板工程，确保顺利通过技术评估和考核鉴定，成为我省首个“国家卫生城市”。

――加强城市社区建设。充分调动社会团体、企事业单位以及广大居民的积极性，大力推进社区建设，推动社区资源共享。按照责、权、利统一的原则，着力完善城市基层社会管理和服务体制，积极解决社区居委会办公场地和人员待遇问题，引导社区中介组织有序发展、规范运作，依托社区广泛开展和谐创建活动，实现政府行政管理和社区自我管理的有效衔接。

四、积极统筹城乡发展，大力推进新农村建设

要继续加大财政支农力度，坚持以文明生态村为综合创建载体，以加快农业现代化进程为重点，大力实施“双增双提一转变”发展战略（农民增收，农业增效；提高土地产出率，提高农产品质量安全；由传统农业向现代农业转变），积极推进新农村建设。

――加快农业现代化进程。立足城市需求调整农业结构，以扶龙头、带基地、打品牌、拓市场、靠科技、抓培训、建设施、打基础为主要手段，着力提高土地产出率，促进农业增效。在生产方式方面，要围绕设施农业，加快推行“龙头企业（行业协会）+农民专业合作社”等合作模式，积极引导龙头企业与农户实行产销联合，使设施农业向基地化、集约化、标准化生产和规模化经营方向发展，成为农民致富的主导产业。在市场开拓方面，要不断完善农业市场体系，加快“三电合一”农业信息服务项目建设，抓好农产品包装宣传，树立三亚农产品品牌，继续积极开拓岛外市场。在产品选择方面，要进一步加大兰花等热带花卉产业发展力度，有选择地发展经济效益较好的苗木产业；要加快发展无公害农产品，强化农业投入品管理，加强农产品质量安全保障，重点围绕冬季瓜菜、热带水果等特色优势产品抓好供港澳果蔬基地建设；要充分利用无疫区的有利条件，抓好良种猪繁育基地和湾坡鸭苗孵化场建设。在农业科技方面，要加快科技成果引进和推广，进一步健全农村科技服务体系，抓好农技培训和技术咨询，努力培育现代农民。在基础设施方面，要加快大隆灌区工程建设，对安罗、抱古等水库部分灌区渠道实施维修改造，实施抱古、岭渣等10宗水库除险加固工程，进一步改善农村生产条件。

――大力促进农民增收。认真落实农机具购置补贴、沼气池建设补贴、兰花种植补贴等支农惠农政策，增加农民政策补贴收入。积极发展乡镇企业，培育农村二、三产业，开发乡村旅游项目，支持“旅游示范村”、“旅游示范户”发展。着力抓好农民的职业技能培训，加大农村富余劳动力转移力度，把安排就业作为与开发商签约的前提条件之一，努力扩大企业吸纳本地农民就业比重，积极发展“打工经济”，争取实现农村劳动力转移就业8000人，推动农民增收途径多元化发展。

――进一步改善农村生活条件。要着力抓好60个新农村示范点建设，加快推进农村路网、电网、民房改造、饮水安全、改厕、沼气池、环保设施等工程建设。要大力发展生态农业，推广种-养-沼气生态农业模式，新建农村（含学校）沼气池3000户。要全力加强扶贫攻坚工作，力争尽早实现19个贫困村的脱贫目标。

――积极推进农村综合配套改革。探索推动农村金融机构发展，稳妥推进农村信用社改革，推行小额信贷、农户联保贷款等贷款方式，不断完善农业保险。规范和健全农村土地承包经营权流转市场。积极完善村级组织管理体制，逐步建立社会主义新农村建设的长效机制。

五、不断加强文化建设，积极促进文化繁荣

我们要继续加强和谐文化建设，进一步挖掘城市文化内涵，大力发展文化事业，推动社会主义文化大发展大繁荣。

――加强和谐文化建设。树立以“八荣八耻”为主要内容的社会主义荣辱观，倡导爱国、敬业、诚信、友善等道德规范。积极开展社会公德、职业道德教育，加强青少年思想道德建设，着力营造诚实守信的社会环境。加大科普工作力度，倡导科学、健康、文明的生活方式，大力推行城乡居民文明规范。充分发挥广播电视、报刊、互联网站等媒体的舆论导向作用，提高舆论引导水平。积极开展城乡社会志愿服务活动，加强心理健康教育和咨询。

――促进文化事业繁荣。完成文化体制改革试点工作，积极引导社会力量参与文化事业发展。加快海滨影剧院改造，积极推进群众艺术馆、博物馆等场馆建设。进一步加强对崖城孔庙、落笔洞遗址等历史文物的保护、管理、开发和利用，尽快理顺大小洞天管理体制，探索开发历史文化旅游。充分挖掘、大力保护和积极传承黎、苗、回等少数民族文化以及民俗文化，积极推进三亚黎族文化博览村建设工作。尽快建成两区综合文化中心，积极推动社区文化体育广场建设，做好文化下乡和文体进社区工作，结合奥运开展全民健身活动，继续推进广播电视村村通工程和有线数字电视转换工作，丰富人民群众特别是农村、偏远地区群众以及进城务工人员的文体生活。

六、始终坚持以人为本，全心全意改善民生

我们要积极推动教育、医疗等社会事业加快发展，切实抓好就业、社保工作，不断提高人民生活质量，保持社会稳定。

――优先发展教育事业。进一步加大教育投入，逐步使财政性教育经费占GDP比例达到4%。加快教育资源合理布局和结构调整，促进义务教育均衡发展；进一步加强教育基础设施特别是农村教育基础设施建设，完成育才青法小学、雅林小学等扶贫攻坚项目建设；积极推进市教师进修学校二期建设，实施名校长、名师“引培工程”和教师读书工程，加大教师培训力度，进一步提升师资水平；继续开展城镇学校和农村薄弱学校教师支教、交流工作，适当提高教师尤其是农村边远地区教师待遇；继续加强助学体系建设，积极做好城区特困家庭子女和进城务工人员子女接受义务教育的工作。扩大优质高中教育规模，强化薄弱高中建设，逐步创造条件普及高中阶段教育。抓好市中等职业技术学校二期建设，继续实行中等职业技术教育“三免一补”政策，探索推行教育扶贫移民；深化职教教学改革，积极推行工学结合、校企合作，并针对企业在岗职工和农村富余劳动力开展职业技术培训。推进三亚学院三期、三亚航空旅游职业学院三期、琼州学院三亚校区、三亚城市职业学院和三亚理工职业学院等院校建设，力争洛桑管理学院、长江商学院分院等项目落户三亚，促进高等教育发展。

――加快医疗卫生等社会事业发展。继续推进市人民医院、市中医院项目建设，做好妇幼保健院和市疾病预防控制中心整体搬迁前期工作，加快农垦三亚医院传染病区设备配套。完成12个社区卫生服务站建设，增强社区卫生服务能力，实行社区医疗卫生服务机构与市级医疗机构双向转诊制度，探索推动社区首诊制度试点。完成村卫生室建设任务，继续抓好农村卫生院建设和设备配置等工作，认真开展城市支援农村卫生工作。加快推进公立医疗机构内部运行机制和人员分配制度改革，进一步深化医院采购制度和医疗服务价格改革，规范医疗采购行为，科学确定限价病种、收费标准，完善医疗收费及药品价格公开和查询制度，着力解决看病贵问题。加强药品供应和安全监管网络建设，保证医疗安全。进一步稳定低生育水平，加强性别比综合治理。广泛开展国防教育，大力支持国防建设，加强国防动员和民兵预备役工作，继续深入开展拥军优属、拥政爱民活动，进一步提高双拥共建质量。继续做好妇女儿童、老龄工作，外事侨务、民政、统计、档案、修志、科协、侨联、残联、新闻等工作也要取得新进展。

――进一步加强就业工作。继续实施积极的就业政策，力争2008年新增就业岗位15000个以上，城镇登记失业率控制在3%以内。加快户籍和劳动就业制度改革，做好新人力资源市场设施配置和启用工作。加快人才劳动力市场信息化建设，进一步健全就业援助制度，重点促进“零就业家庭”成员、“4050”等就业困难人员就业。进一步完善和落实税费减免、小额担保贷款、资金奖励以及社会保险补贴等优惠政策，鼓励自主创业和自谋职业。加强规范和协调劳动关系的工作，依法维护劳动者尤其是农民工的合法权益。

――建立健全覆盖城乡居民的社保体系。积极推进企事业单位离退休人员社会化管理服务工作，促进城镇职工基本养老保险制度规范化，确保养老金按时足额发放，继续探索推行农村养老保险制度。进一步完善城镇居民基本医疗保险和新型农村合作医疗制度，切实提高定点医疗机构服务水平，建立健全医疗便利报销制度。进一步完善城乡低保工作，提高低保标准，建立健全新型社会救助体系，将进城务工人员纳入社保体系。继续探索失地农民保障问题，考虑通过发展物业经营、入股分红等方式增加农民财产性收入。大力发展社会福利事业，促进红十字事业发展，推进三亚民政服务中心和残疾人综合服务中心等设施建设。

――努力提高城镇居民收入水平。根据国家统一部署，积极稳妥地进行公务员工资改革，落实公务员津贴补贴。企事业单位要适当提高职工的工资性收入水平，落实职工带薪年休假制度。切实落实最低工资规定，逐步提高最低工资标准。强化市场物价监管，特别是要提高食品、液化石油气、农资等与群众利益密切相关的商品价格监测水平，努力保持价格总体水平基本稳定。建立健全低收入群体生活消费品价格上涨动态补贴和临时救助制度，确保他们的基本生活不受影响。

――维护社会安定团结。积极推进平安三亚建设，进一步加大社会治安基础投入；加强社会治安综合治理，严厉打击各种有组织犯罪、黑恶势力犯罪和经济犯罪活动，继续深入开展打击“两抢一盗”和毒品犯罪等专项行动；大力实施社区和农村警务战略，全面建设治安监控网络，进一步完善社会治安防控体系。积极完善领导干部接待群众制度，进一步落实信访责任制，建立健全社会利益协调机制和社会矛盾纠纷调处机制，预防和妥善处置群体性事件，耐心解决人民群众的困难和问题。严格落实安全生产责任目标管理制，不断加大安全隐患排查和治理工作力度，坚决遏制重特大安全事故的发生，保障人民群众生命财产安全。建立健全重大公共安全事件的预警、应急、救援体系，加快公共安全抢险救援应急中心建设，增强政府应对突发事件的能力。全面贯彻党的民族宗教政策，加大对少数民族地区发展的支持力度，防止境外敌对势力和非法宗教势力的渗透。

七、认真做好节能减排工作，进一步加强生态环境保护

我们要按照建设资源节约型社会和环境友好型社会的要求，切实落实节能减排工作，加强生态环境保护，实现可持续发展。

――抓好节能减排工作。加快推进红沙污水处理厂升级改造、鹿回头区污水处理、三亚湾新城污水处理等污水处理项目以及亚龙湾冰蓄冷区域供冷系统、市区污水管网系统改造等项目建设，年内要实现中水利用。做好第一次全国污染源普查，严格执行节能减排目标责任制，强化节能降耗和污染减排指标约束。加大项目环保审批力度，严格控制新（扩）建高耗能及使用高污染燃料的项目，环保设施与建设项目主体工程必须同时设计、施工、投产。强化排污许可证审批管理，主要排污单位的持证率要达到90%以上。突出抓好制冰、化工、水产品加工、木材加工等重点行业、重点企业的节能减排工作，实施重点企业节能环保工程，禁止使用实心粘土红砖，鼓励使用新型墙体材料，鼓励利用太阳能、风能等清洁能源，2008年万元GDP能耗要下降2.3%以上。

――大力推进生态市建设。组织编制完成《三亚生态市建设总体规划》，加快完善饮用水源地环境保护规划体系，抓好封山育林、退耕还林、退塘还林等工作，积极推进城市环境综合治理、重点污染源治理等生态保护工程，重点实施对三亚湾海滩泥化和海岸侵蚀的综合治理，加大榆林红沙港、三亚渔港的海洋环境整治力度。加快实施沿海基干林带恢复建设工程，继续推进红树林公园、月川新城区湿地公园、三亚西河槟榔河段治理项目。进一步完善环保基础设施，加快推进垃圾收集转运系统、生活垃圾填埋场以及医疗废物处理中心等项目建设。着力提升环境监测水平，制定应急监测预案，继续开展环保专项行动，进一步加大对噪声、汽车尾气等污染的治理力度。

八、加强政府自身建设，提高政府服务效能

面对三亚发展的新形势和新任务，我们必须按照科学执政、民主执政、依法执政对政府的要求，进一步加快政府职能转变，不断加强政府自身建设，打造高效能的服务型政府。

――切实加强制度建设。“用制度管权、按制度办事、靠制度管人”，是提升工作效率和防止腐败滋生的有效途径。一是要创新资源配置制度。进一步转变政府角色，充分发挥市场的配置作用，在土地、海域、城市公共资源等方面大力推行公开招标拍卖出让制度，优化资源配置，实现资源效益最大化。二是要继续深化行政审批制度改革。进一步简化审批制度，加强审批规范化、程序化建设，严格落实工作时限要求，通过完善制度管住工作人员，净化审批行为。三是要完善法律法规应用制度。加快法治政府建设，切实推进依法行政，建立健全重大决策公示、听证制度和政府法律顾问制度，深化行政执法体制改革。四是要加快建立健全职权责统一的制度。科学划分市、镇职责，合理配置管理权限，按照事权与财力相匹配原则，进一步理顺市、镇财政关系。按照精简原则合理配置编制，理顺部门分工，明确职能、权限和责任。科学设置岗位，将责任和权力具体到每一个岗位、每一个人。

――强化行政效能监督。进一步完善决策目标、执行责任、考核监督体系，建立健全科学的综合绩效考评制度，切实落实行政过错追究制和行政首长问责制。进一步加大行政监察和审计监督力度，着力规范行政审批、执法检查、收费征税、行政强制、立案处罚等行政权力的运作。充分发挥社会舆论监督作用，促进政府与社会、干部与群众的互动，欢迎社会各界对政府工作进行监督。进一步加大反腐工作力度，严肃查处以权谋私、权钱交易、“吃拿卡要”、与民争利等违法乱纪行为。

――加强民主法制建设。广泛开展普法教育，引导全社会学法、知法、守法、用法，不断提高公民法制意识和法律素质。自觉向人大及其常委会报告工作，接受人大的法律监督和工作监督，支持和保证人大及其常委会依法行使各项权力。充分尊重人民政协以及各民主党派在参政议政中的作用，自觉接受其民主监督。认真做好人大议案、建议和政协提案的办理工作。积极加强同民主党派、工商联以及无党派人士的联系，认真听取他们的意见。支持工会、共青团、妇联等人民团体依法参与社会管理和公共服务，充分发挥其桥梁纽带作用。积极推进基层民主政治建设，完善政务公开、村务公开等制度，切实保证人民群众的知情权、参与权、表达权和监督权。

各位代表！三亚的发展已经进入了一个新的关键时期，成绩令人鼓舞，宏图已经绘就，明天更加美好。让我们更加紧密地团结在以胡锦涛同志为总书记的党中央周围，在市委的坚强领导下，高举中国特色社会主义伟大旗帜，以邓小平理论和“三个代表”重要思想为指导，全面贯彻党的十七大精神，深入贯彻落实科学发展观，团结一致，开拓创新，锐意进取，为实现三亚又好又快发展而努力奋斗！
